# Isobolographic analysis of co-administration of two plant-derived antiplasmodial drug candidates, cryptolepine and xylopic acid, in *Plasmodium berghei*

**DOI:** 10.1186/s12936-018-2283-8

**Published:** 2018-04-04

**Authors:** Elvis O. Ameyaw, Kodwo B. Asmah, Robert P. Biney, Isaac T. Henneh, Phyllis Owusu-Agyei, James Prah, Arnold D. Forkuo

**Affiliations:** 1 Department of Biomedical Sciences, School of Allied Health Sciences, College of Health and Allied Sciences, University of Cape Coast, Cape Coast, Ghana; 2 Department of Pharmacology, School of Medical Sciences, College of Health and Allied Sciences, University of Cape Coast, Cape Coast, Ghana; 3 University of Cape Coast Hospital, Cape Coast, Ghana; 4 Department of Pharmacology, Faculty of Pharmacy and Pharmaceutical Sciences, College of Health Sciences, Kwame Nkrumah University of Science and Technology, Kumasi, Ghana

**Keywords:** Malaria, Artemisinin combination therapy, Parasitemia, Cryptolepine, Xylopic acid

## Abstract

**Background:**

Increasing resistance to current anti-malarial therapies requires a renewed effort in searching for alternative therapies to combat this challenge, and combination therapy is the preferred approach to address this. The present study confirms the anti-plasmodial effects of two compounds, cryptolepine and xylopic acid and the relationship that exists in their combined administration determined.

**Methods:**

Anti-plasmodial effect of cryptolepine (CYP) (3, 10, 30 mg kg^−1^) and xylopic acid (XA) (3, 10, 30 mg kg^−1^) was evaluated in *Plasmodium berghei*-infected male mice after a 6-day drug treatment. The respective doses which produced 50% chemosuppression (ED_50_) was determined by iterative fitting of the log-dose responses of both drugs. CYP and XA were then co-administered in a fixed dose combination of their ED_50_s (1:1) as well as different fractions of these combinations (1/2, 1/4, 1/8, 1/16 and 1/32) to find the experimental ED_50_ (Z_exp_). The nature of interaction between cryptolepine and xylopic acid was determined by constructing an isobologram to compare the Z_exp_ with the theoretical ED_50_ (Z_add_). Additionally, the effect of cryptolepine/xylopic acid co-administration on vital organs associated with malarial parasiticidal action was assessed.

**Results:**

The Z_add_ and Z_exp_ were determined to be 12.75 ± 0.33 and 2.60 ± 0.41, respectively, with an interaction index of 0.2041. The Z_exp_ was significantly (*P *< 0.001) below the additive isobole indicating that co-administration of cryptolepine and xylopic acid yielded a synergistic anti-plasmodial effect. This observed synergistic antiplasmodial effect did not have any significant deleterious effect on the kidney, liver and spleen. However, the testis were affected at high doses.

**Conclusion:**

The co-administration of cryptolepine and xylopic acid produces synergistic anti-malarial effect with minimal toxicity.

## Background

Every 2 min a child dies due to malaria in sub-Saharan Africa. It is estimated that 88% of the cases and 90% of the malaria deaths reported in 2015 were in the WHO Africa region while most deaths (70%) were in children under 5 years of age [1]. Globally, an estimated 3.2 billion people are at risk of the disease with 214 million new cases of malaria and a corresponding mortality of 438,000 recorded in only the year 2015 [1].

The well-known use of chloroquine as a monotherapy for malaria treatment is no longer effective in most endemic areas. Combination therapy has therefore emerged as the best practical solution to overcome the resistance of select strains to conventional first-line anti-malarial drugs owing to increased efficacy and reduced toxicity. Anecdotal study records suggest that artemisinin-based combinations, which are the mainstay drugs for the management of uncomplicated malaria, have showed increased treatment failure rates over 10% in Angola, Burkina Faso, the Gambia, Ghana, Malawi, the Niger, Nigeria and Zimbabwe [2]. This situation is likely to increase as a result of increased drug pressure. It is, therefore, very important to search for novel, affordable, and efficient synergistic combinations to augment the antiplasmodial activity of existing combination drugs.

Due to the crucial role that plant-derived compounds have played in drug discovery and development for the treatment of several diseases, the isolation of new bioactive compounds from medicinal plants based on traditional use or ethno medical data appears to be very promising approach. According to the World Health Organization (WHO) [1], studies have documented over 1200 plant species from 160 families used in the treatment of malaria. *Cryptolepis sanguinolenta* (Apocynaceae) and *Xylopia aethiopica* (Annonaceae) are two medicinal plants commonly used by Ghanaian herbal practitioners to treat malaria [3, 4]. Cryptolepine, the indoquinoline alkaloidal constituent of *Cryptolepis sanguinolenta* has been reported to be responsible for the anti-hyperglycaemic [5, 6], anti-inflammatory [7], antibacterial [8] and antiplasmodial [9, 10] activities of the plant. In the unripe fruits of *Xylopia aethiopica*, the kaurene diterpene, xylopic acid, which occurs as the major constituent has been reported to possess antipyretic and anti-malarial [11], analgesic [12, 13] and anti-depressant [14] properties among others.

The antiplasmodial effects of both cryptolepine and xylopic acid have been demonstrated in two separate studies [11, 15]. In the present study, we tested the hypothesis that these two plant-derived compounds have synergistic antiplasmodial effects was tested and the toxicity profile of co-administering the two compounds also assessed.

## Methods

### Extraction of cryptolepine

**Fig. 1:**
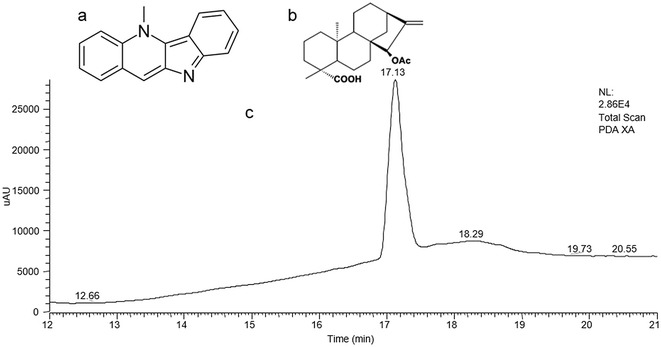
Chemical structure of **a** cryptolepine, **b** xylopic acid and **c** HPLC fingerprint of xylopic acid

Cryptolepine (Fig. 1a) was a gift from Professor Colin W. Wright. It was isolated from powdered dried roots of *Cryptolepis sanguinolenta* as described by previous studies [16]. The identity of isolated CYP was confirmed by the mass spectrometry, thin layer chromatography (TLC), high performance liquid chromatography (HPLC) and the melting point determination similar to an earlier report [15].

### Extraction of xylopic acid

Fresh unripe fruits of *Xylopia aethiopica* were collected from Kwame Nkrumah University of Science and Technology Botanic Gardens. The fruits were dried for 21 days and pulverized into a fine powder with a hammer mill. A quantity of the powdered material was macerated with petroleum ether (40–60 °C) and allowed to stand for 3 days. The extract was concentrated with a rotary evaporator at a temperature of 50 °C and ethyl acetate added to the concentrate to facilitate the crystallization of xylopic acid. Xylopic acid crystals formed after the concentrate had been allowed to stand for 72 h. This was washed with petroleum ether at 40–60 °C repeatedly to remove all unwanted materials. The resulting crude xylopic acid was finally purified in 96% ethanol by recrystallization as previously described [12, 13, 17]. The purity of xylopic acid (Fig. 1b) was confirmed by HPLC analysis to be 98% w/w pure (Fig. 1c).

### Animals

Male ICR mice (20 ± 5 g) were purchased from Noguchi Memorial Institute for Medical Research, University of Ghana, Legon, Ghana and randomly assigned into groups of five in stainless steel cages with soft wood shaving as bedding, under ambient laboratory conditions. They were fed a normal commercial pellet diet (AgriCare, Kumasi) and water given ad libitum. The cages were housed in the animal facility of the Department of Biomedical and Forensic Sciences, University of Cape Coast (UCC). All experiments were carried out in accordance with guidelines of the University of Cape Coast Ethics Committee and NIH guidelines for the care and use of laboratory animals.

### Rodent parasite *Plasmodium berghei* NK65

The rodent parasite, chloroquine-sensitive strain of *Plasmodium berghei* NK-65 was obtained from Noguchi Memorial Institute for Medical Research, University of Ghana and maintained alive by continuous intraperitoneal passage in mice after every 6 days [18]. The re-infected mice were kept in the animal house of the Department of Biomedical and Forensic Sciences at University of Cape Coast.

### Drugs, chemicals and reagents

Combined artemether/lumefantrine tablets (20/120 mg) were obtained from Novartis Pharma AG Basel, Switzerland. Sodium chloride, NaH_2_PO_4_·H_2_O, Na_2_HPO_4_, Triton X-100, Giemsa stain, ethyl acetate, liquid paraffin, chloroform, 96% ethanol, absolute methanol (acetone free), glycerol, tween 20, petroleum ether, ammonium hydroxide and conc. HCl were all purchased from Sigma-Aldrich, St. Louis, MO, USA. Normal saline (0.9% NaCl) was purchased from Intravenous Infusions, Koforidua, Ghana.

### Inoculation of parasite

A total inoculum concentration of 6.3 × 10^7^ of *P. berghei* parasitized erythrocytes ml^−1^ was prepared by determining the parasitaemia of *Plasmodium berghei*-infected mice before diluting with EDTA-phosphate buffered saline (PBS) to obtain each of the targeted concentrations and subsequently washed with PBS [11]. 35 mice were each inoculated with 0.20 ml of 1 × 10^6^ *P. berghei.*

### Determination of animal body weight

To determine the effect of CYP, XA and CYP + XA dose combination on body weight during treatment, the weight of each experimental mouse was measured on days 0 and 6 post infection with a sensitive weighing balance (Mettler Toledo, Switzerland) and the change in weight post treatment calculated.

### Anti-malarial activity

#### In vivo anti-malarial assay of cryptolepine and xylopic acid monotherapies

To confirm the reported antiplasmodial effects of CYP and XA and also compute ED_50_ values for the isobolographic analysis, the individual anti-plasmodial activity of the compounds were assessed. *Plasmodium berghei*-infected mice were randomly assigned to 9 groups (n = 5). The animals in the 9th group were not inoculated and served as a naïve control. Seventy-two hours after *P. berghei* inoculation (day 1), the 8 groups of animals were treated once daily for 5 days *per os* with either xylopic acid (10, 30, 100 mg kg^−1^), cryptolepine (10, 30, 100 mg kg^−1^), artemether/lumefantrine (1.14/6.9 mg kg^−1^) or distilled tween 20 (vehicle) (10 mg kg^−1^). ED_50_ values of CYP and XA was computed for the isobolographic studies by iterative curve fitting of log-dose responses of CYP and XA used in the monotherapy experiment and determining the fitted midpoints. The mean survival time of the mice in each treatment group was determined over a period of 30 days.

#### In vivo isobolographic assessment of cryptolepine–xylopic acid co-administration on *Plasmodium berghei*-induced malaria

To evaluate the anti-malarial property of cryptolepine–xylopic acid co-administration (CYP/XA) on established *P. berghei* infection, thirty-five male mice were each inoculated with 0.20 ml of 1 × 10^6^ *P. berghei* and then randomly assigned to nine groups (n = 5). Seventy-two hours later (day 1), they then received either the ED_50_ (equi-effective doses) or fixed ratio (1:1) combinations of fractions of the respective ED_50_ values of (15 + 11 mg kg^−1^), (7.5 + 5.5 mg kg^−1^) (3.8 + 2.8 mg kg^−1^), (1.9 + 1.4 mg kg^−1^), (0.9 + 0.7 mg kg^−1^) ED_50_(CYP + XA), [ED_50_(CYP + XA)/2, ED_50_(CYP + XA)/4, ED_50_(CYP + XA)/8 and ED_50_(CYP + XA)/16, respectively. Negative and positive control animals received vehicle (10 ml kg^−1^) and artemether/lumefantrine (1.14/6.9 mg kg^−1^), respectively.

#### Parasitaemia and percentage chemosupression determination

Daily parasitaemia of each mouse was determined post-inoculation by collecting three drops of blood from the tail vein. The blood was subsequently smeared onto a microscope slide to make a thin film. The smears were fixed in absolute ethanol, stained with 10% Giemsa stain, and examined microscopically at magnification of ×100. The parasitaemia was determined by counting infected erythrocytes in hundred fields, divided by the total erythrocytes in the hundred fields and then multiplied by hundred. This was calculated as follows:

$$\% \;Parasitaemia = \frac{Number\,of\,P.\,berghei - infected\,erythrocytes}{Total\,number\,of\,erythrocytes} \times 100$$

The percentage (%) inhibition or chemosupression was calculated using the following formula:

$$\% \,Inhibition = \frac{{\left( {Mean\,parasitemia\,of \,negative\,control} \right) - \left( {Mean\,parasitemia\,of\,test\,drug} \right)}}{Mean\,parasitemia\,of\,negative\,control} \times 100$$

### Effect of cryptolepine/xylopic acid co-administration on selected organs

To assess the toxicological or beneficial effect of the compounds, two male animals from each treatment group were randomly selected and humanely sacrificed on the 6th day post infection. The liver, spleen, kidney and testis were harvested and samples washed separately with 0.9% NaCl. They were fixed in 10% neutral phosphate buffered (with NaH_2_PO_4_) formalin for 24 h, embedded in paraffin, and 8 µm sections cut on a microtome (Bright 5040, Bright instrument company Ltd., England) and processed for routine haematoxylin–eosin (H&E) staining.

### Data analysis

Graph Pad Prism for windows version 6.01 (Graph Pad Software, San Diego, CA, USA) was used for all statistical analysis with P < 0.05 considered statistically significant. All data are presented as the mean ± SEM. One-way ANOVA was applied to data analysis followed by Tukey’s HSD (honest significant difference) test.

An isobologram consisting of the ED_50_ of XA on the ordinate and ED_50_ of CYP on the abscissa, connected with a line of additivity was constructed. For each drug combination, the ED_50_ (experimental) and its associated SEM was determined by linear regression analysis of the log dose–response curve (and compared by a T-test to a theoretical additive ED_50_ i.e. Z_add_). The Z_add_ was calculated as follows:

$$Z_{add} = \left( f \right)ED_{50}\,of\,CYP + \left( {1 - f} \right)ED_{50}\,of\,XA$$

where f is the fraction of each component in the mixture/combination while the variance (Var) of Z_add_ was calculated as:

$$Variance\,of\,Z_{add} = f2\left( {Var ED_{50}\,of\,CYP} \right) + \left( {1 - f} \right)2VarED_{50 }\,of\,XA$$

From these variances SEMs were calculated and resolved according to the ratio of the individual drugs in the combination. A supra-additive or synergistic effect was defined as the effect of a drug combination that was higher and statistically different (ED_50_ significantly lower) than the theoretically calculated equieffect of a drug combination in the same proportion.

## Results

### Mean body weight of animals

**Fig. 2:**
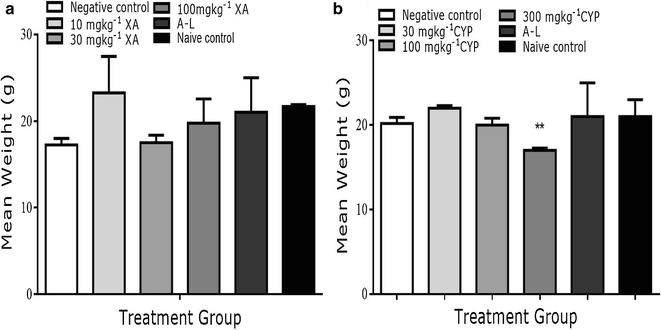
Mean body weight of xylopic acid (**a**) and cryptolepine (**b**)-treated animals in the antiplasmodial monotherapy assay. Data represented as mean ± SD (n = 5)

There were no significant changes in mean body weight of XA-treated (Fig. 2a) mice and all CYP + XA-treated animals (Table 1). Only mice treated with CYP 300 mg kg^−1^ (Fig. 2b) exhibited a significant change in body weight compared to naïve control (Fig. 2a, b).

**Table 1:** Mean body weight analysis of *Plasmodium berghei*-infected mice before and after co-administration with xylopic acid + cryptolepine

Treatment	W_0_ (g)	W_6_ (g)	% weight diff.
CYP + XA	19.15 ± 0.67	19.49 ± 0.66	1.79
CYP + XA	19.17 ± 1.10	19.61 ± 1.12	2.20
CYP + XA	19.66 ± 1.27	20.01 ± 1.32	1.77
CYP + XA	20.05 ± 2.43	20.30 ± 2.48	1.27
CYP + XA	20.91 ± 1.09	20.95 ± 1.08	0.21
Vehicle	19.49 ± 1.32	19.00 ± 1.29	− 2.52
ART + LUM	21.64 ± 1.43	22.18 ± 1.37	2.48
Naive	20.03 ± 0.58	21.30 ± 0.30	6.33

Data is presented as mean ± SEM, n = 5, P < 0.05, W0: mean weight pre-treatment on 1st day, W_6_: mean weight post-treatment on sixth day

### Anti-malarial assessment of cryptolepine and xylopic acid mono therapies and co-administration on established *P. berghei* infection in mice

**Fig. 3:**
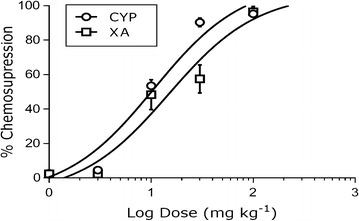
Log dose–response curve for *P. berghei*-infected mice co-administered daily with xylopic acid (XA) + cryptolepine (CYP) over 5 days. Data is presented as mean ± SEM (n = 5)

The parasitaemia (day 1 of treatment), chemo suppression (final day of treatment) and survival days for the various treatment groups are presented on Table 2. The ED_50_s of cryptolepine and xylopic acid were 10.79 ± 0.07 and 14.83 ± 0.10 mg kg^−1^ respectively. In this investigation, cryptolepine was thus 1.4 times more potent than xylopic acid (Fig. 3).

**Table 2:** Parasitaemia, chemo suppression and survival days of mice treated with different combination concentrations of cryptolepine/xylopic acid for 5 days

Treatment	Dose (mg kg^−1^)	% parasitaemia	% chem supp	Survival
		Day 1	Day 6	Days
Vehicle	0.50 ml	19.2 ± 4.1	–	9.2 ± 0.8
CYP + XA	15 + 11	21.5 ± 3.3	78.2 ± 2.9	18.0 ± 1.4
CYP + XA	7.5 + 5.5	19.2 ± 2.7	67.0 ± 0.1	15.3 ± 0.6
CYP + XA	3.8 + 2.8	20.1.1 ± 2.0	65.5 ± 2.6	15 ± 1.8
CYP + XA	1.9 + 1.4	18.5 ± 3.1	50.6 ± 2.4	13.3 ± 1.4
CYP + XA	0.9 + 0.7	18.2 ± 1.3	40.0 ± 1.5	14.0 ± 2.5
ART + LUM (positive control)	1.14 + 16.9	21.3 ± 57.9	90.3 ± 1.7	25.2 ± 1.3

Values are mean ± SEM

### Isobolographic analysis of antiplasmodial effects of CYP and XA co-administration

**Fig. 4:**
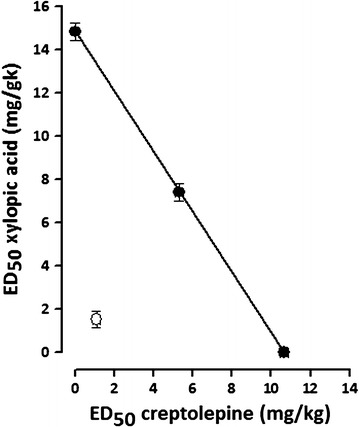
Isobologram of the co-administration of cryptolepine and xylopic acid. Filled circles indicate theoretical ED_50_ ± SEM while open circle indicate experimental ED_50_ ± SEM. The line of additivity connects the ED_50_ of Xylopic acid on the abscisa to that of cryptolepine on the ordinate

The theoretical ED_50_ (Z_add_) of cryptolepine and xylopic acid co-administration was obtained as 12.75 ± 0.33 whereas the experimental ED_50_ (Z_exp_) of the mixture was 2.60 ± 0.41. The Z_exp_ (open circle) lay significantly below the line of additivity as well as the Z_add_ (closed circles) on the isobologram indicating synergism (Fig. 4). The degree of interaction calculated as the interaction index was 0.2041 (Table 3).

**Table 3:** Theoretical (Z_add_) and experimental (Z_exp_) ED_50_ ± SEM values of xylopic acid and cryptolepine co-administration in the anti-malarial test

ED_50_s (XA/CYP: 1:1)	Anti-malarial activity
Z_add_ (mg kg^−1^)	12.75 ± 0.33
Z_exp_ (mg kg^−1^)	2.60 ± 0.41
Interaction index	0.2041

### Effect of fixed dose combinations of cryptolepine and xylopic acid on selected organs

#### Effects of CYP + XA on kidney

**Fig. 5:**
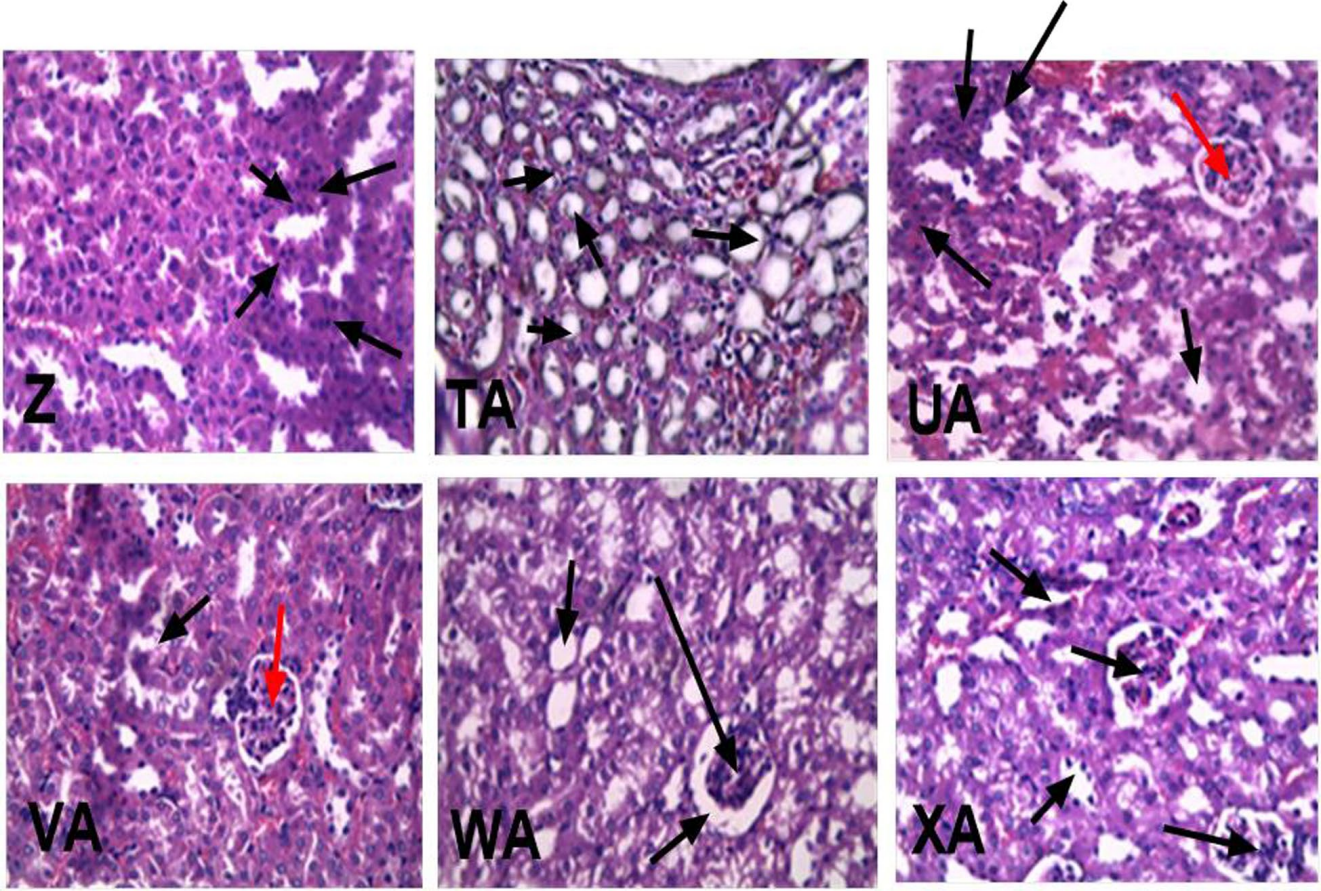
Effects of CYP/XA dose combinations on the kidney. (Z) XA-CYP (15 + 10 mg kg^−1^) (TA) XA-CYP/2 (UA) XA-CYP/4 (VA) XA-CYP/8 (WA) XA-CYP/16 (XA) vehicle-treated

Tissues showed some diminished renal corpuscles. There was also dilation and congestion of renal blood vessels of the negative control mice. The glomerulus of the CYP/XA (1.87 + 1.4 mg kg^−1^) [ED_50_(CYP + XA)/16]-treated mice appeared normal but with dilation and congestion of blood vessels while CYP/XA (0.93 + 0.63 mg kg^−1^) [ED_50_(CYP + XA)/32]-treated mice showed increased capsular space of renal corpuscles with atrophy of glomerulus and vacuolation of cuboidal cells especially near the periphery of the cortex (Fig. 5).

#### Effects CYP/XA on the liver

**Fig. 6:**
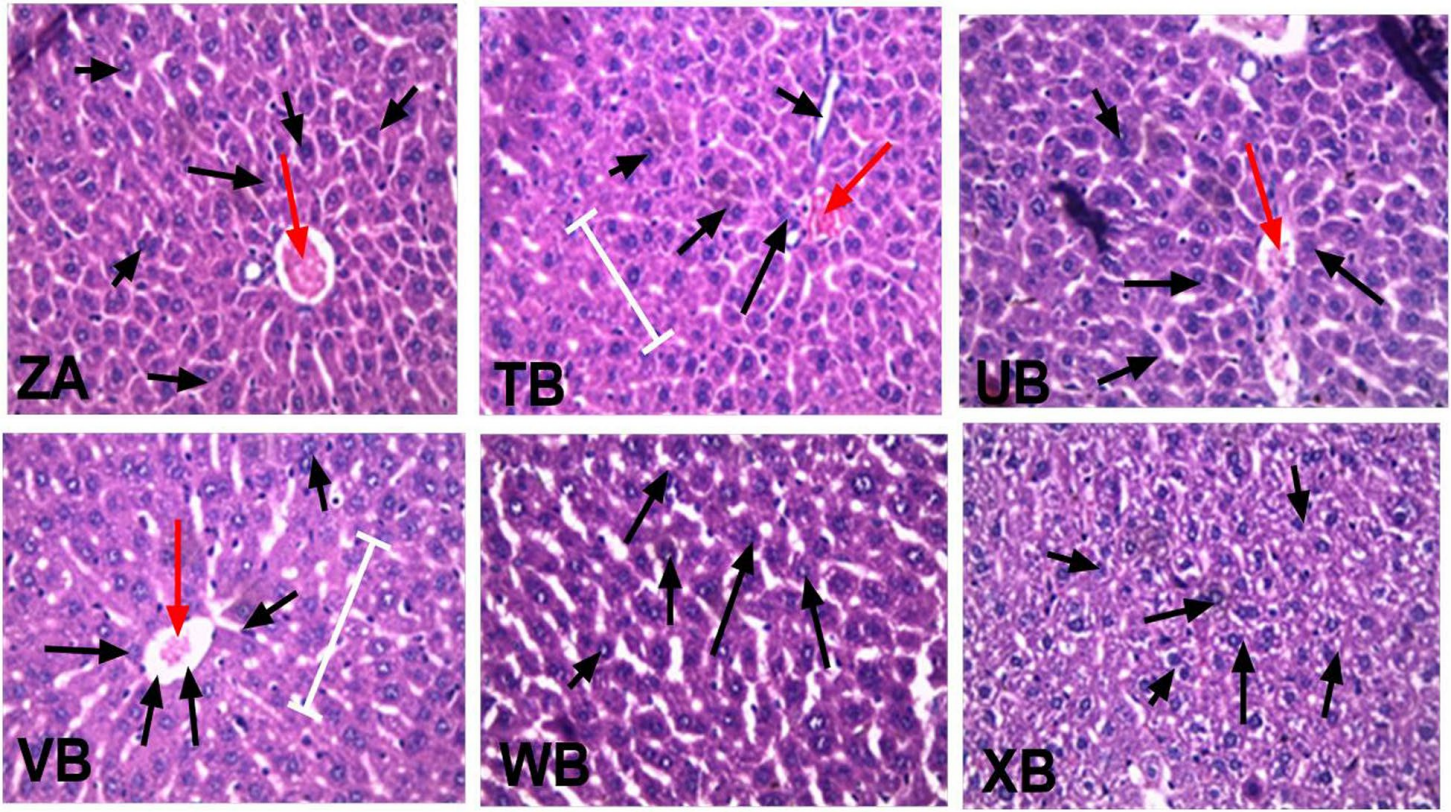
Photomicrograph (×40) showing H&E-stained sections of the liver of cryptolepine (CYP) xylopic acid (XA) co-administration and vehicle-treated *P. berghei* infected mice. (ZA) ED_50_(CYP + XA), (TB) ED_50_(CYP + XA)/2, (UB) ED_50_(CYP + XA)/4, (VB) ED_50_(CYP + XA)/8, (WB) ED_50_(CYP + XA)/16, (XB) vehicle-treated

The liver sections of the experimental animals treated with the different dose combinations of CYP/XA showed relatively intact histological feature similar to that of the animals treated with artemether/lumefantrine. This was in contrast to sections of liver from the vehicle-treated mice which showed severe distortion of the hepatocytes with marked necrosis (Fig. 5). The lower doses ED_50_(CYP + XA)/16 and ED_50_(CYP + XA)/32 treated animals showed signs of high regeneration. Fatty changes were observable mostly within hepatocytes around the central vein. Haemosiderosis and Kupffer cell hyperplasia were mildly observed in the vehicle-treated mice and ED_50_(CYP/XA)/16-treated mice (Fig. 6).

#### Effects of CYP + XA on the spleen

**Fig. 7:**
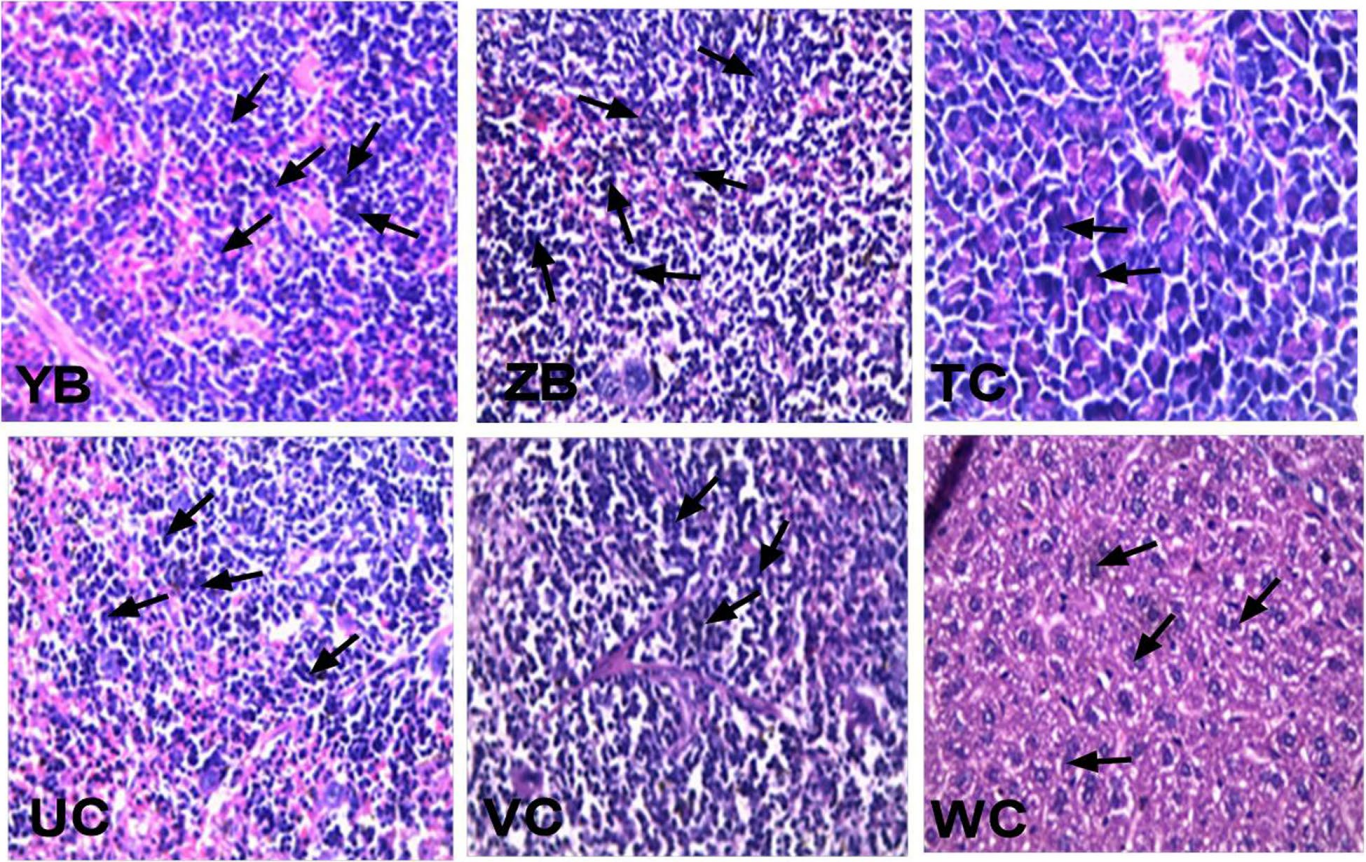
H&E-stained sections of the spleen of xylopic acid (XA) + cryptolepine (CYP) co-administration and vehicle-treated *P. berghei*-infected mice. (YB) ED_50_(CYP + XA) (ZB) ED_50_(CYP + XA)/2, (TC) ED_50_(CYP + XA)/4, (UC) ED_50_(CYP + XA)/8, (VC) ED_50_(CYP + XA)/32 (WC) vehicle-treated (×40)

Mildly edematous spleen was observed with the presence of haemosiderin in the vehicle-treated sections in addition to generalized accumulation and proliferation of macrophages. Splenic architecture was rather normal in CYP + XA co-administered animals except slight macrophage infiltration (Fig. 7).

#### Effects of CYP/XA on the testis

**Fig. 8:**
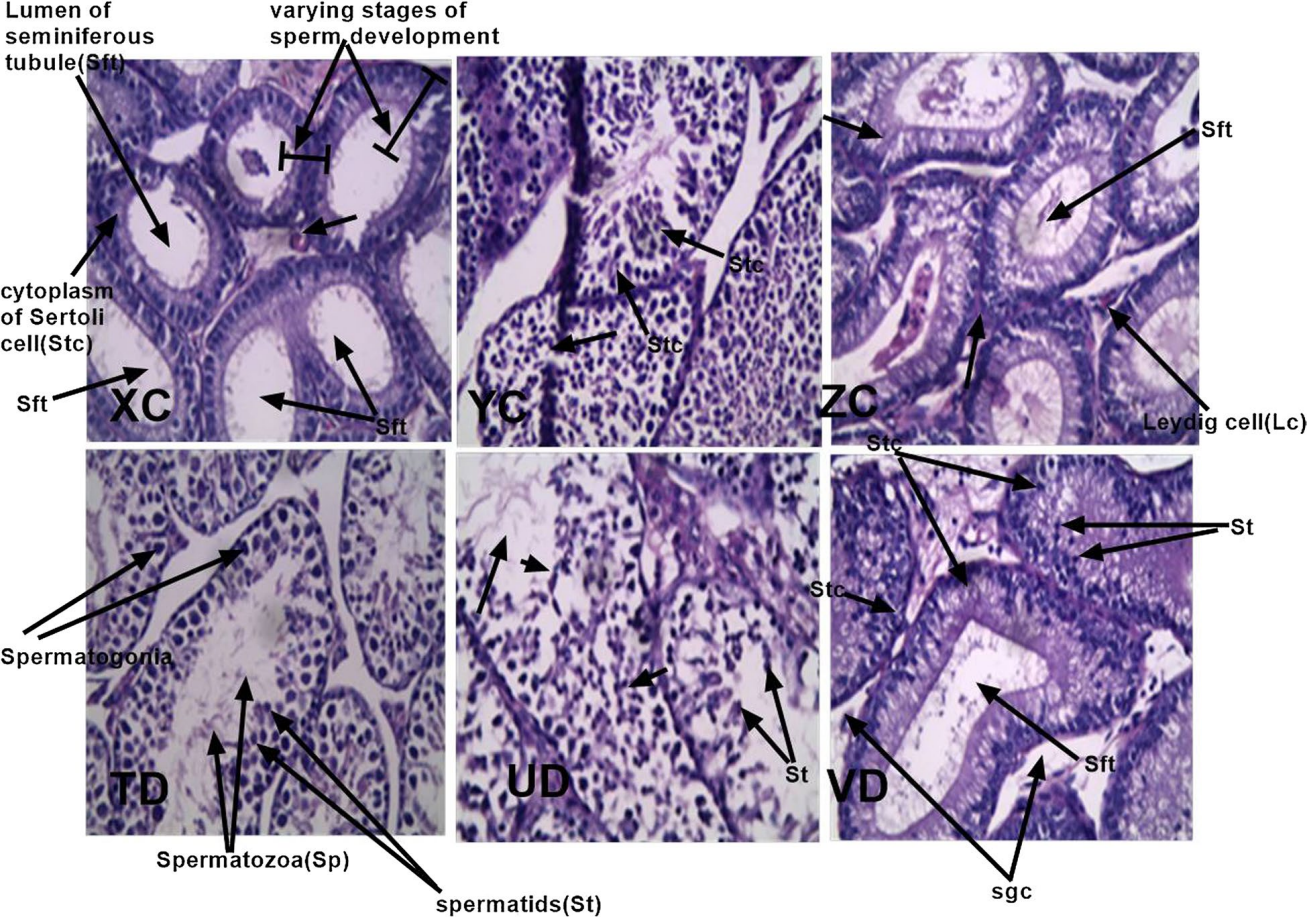
Photomicrograph of H&E-stained sections of testis of xylopic acid (XA)–cryptolepine (CYP) co-administration and vehicle-treated *P. berghei* infected mice. (XC) ED_50_(CYP + XA), (YC) ED_50_(CYP + XA)/2(ZC) ED_50_(CYP + XA)/4, (TD) ED_50_(CYP + XA)/8 (UD) ED_50_(CYP + XA)/16, (VD) vehicle-treated (×40)

Testis histoarchitecture was normal in all treatment groups with intact Sertoli cells and tubules and seminiferous tubules showing the various stages of spermatogenesis and normal spermatids in the negative controls. CY ED_50_(CYP + XA)-treated mice however exhibited some cytotoxic activity due to cleared matured spermatozoa and germ cells in the seminiferous tubules (Fig. 8).

## Discussion

With the emergence of drug resistance, the search and development of new treatment options in treating malaria is the focus on several anti-malarial research groups. The striking success of artemisinin and other anti-malarials of plant origin make the search for new herbal based drugs still relevant [19]. Against the background that combination therapy is preferred to monotherapy in the treatment of malaria, this study examined the effects of combined dose of cryptolepine and xylopic acid. Both compounds isolated from *Xylopia aethiopica* and *Cryptolepis sanguinolenta* have been previously reported as potential drug candidates with antiplasmodial effects [9, 11, 15, 20]. An isobologram is a statistically robust method used to analyse the effect of drug combinations and gives an indication of the nature of interaction when any two drugs are administered together [21, 22].

To ascertain the improved or enhanced antiplasmodial efficacy and potency of cryptolepine and xylopic acid in mice, isobolographic analysis was performed on the two drug candidates. The co-administration of cryptolepine and xylopic acid resulted in significant antiplasmodial activity compared to the vehicle treated animals. The isobologram showed the Z_exp_ laid significantly below the line of additivity (“additive” isobole) and the theoretical Z_add_ of the co-administered drug thus indicating synergism of the anti-malarial effects of the two drug candidates. With an interaction index of 0.2041 which is far less than 1 this is a confirmation of supra-additive or synergistic interaction [23] between cryptolepine and xylopic acid. Thus, the oral co-administration of cryptolepine and xylopic acid in this study produced significant synergistic antiplasmodial effect. The potency of the co-administered agents was significantly higher and lay below the line of additivity and the theoretical potency of the two drug agents in the current test.

The mechanism of anti-malarial action for the co-administered drug is not immediately clear from the present study as both molecules may have acted via their individual respective mechanisms to produce the antiplasmodial synergism or via novel pathway by the admixture.

Cryptolepine exerts its anti-malarial effect by intercalating with the DNA of the Plasmodium parasite in GC-rich sequences thus inhibiting DNA synthesis and stabilizing the topoisomerase II-DNA covalent complex [9, 20]. Alternatively, cryptolepine is reported to offer antiplasmodial activity by preventing haemozoin formation and thereby enhancing free haem toxicity leading to parasite death [9]. Evidence supports the involvement of inhibition of Plasmodium lactate dehydrogenase, an enzyme essential for the anaerobic lifestyle of the parasite in the anti-plasmodial activity of XA [11].

Healthy individuals can relatively detoxify chemicals/drugs when ingested compared to an immune compromised individual. This study tried to determine the potential toxic or ameliorative effects of CYP/XA combinations when malaria infected patients ingest these plant-derived molecules in attempt to treat the infections. It is a common traditional practice in Africa where patients combine *Xylopia aethiopica* and *Cryptolepis sanquinolenta* to treat malaria. The beneficial or detrimental effect of XA/CYP in diseased person may differ from a healthy individual. The potential toxicity of the CYP + XA combination was then evaluated by assessing the integrity of various structures involved in the general malarial parasiticidal action, detoxification and eventually, possible elimination of the test drug in the host. From the study, all the various dose combinations of the cryptolepine/xylopic acid significantly prevented loss of body weight in conformity with previous studies using other medicinal plant products and active drug candidates [24, 25]. Loss of body weight is among the numerous general features of malaria infection which is possibly due to disturbed metabolic function and hypoglycaemia associated with malaria parasite infection [26]. The loss of body weight observed in the highest dose combination may be due to the cytotoxicity nature of cryptolepine which has been observed in other works [6]. An ideal anti-malarial agent is, therefore, expected to prevent body weight loss in infected mice due to the rise in parasitaemia.

During malaria infection, matured merozoites exit the hepatocyte by exocytosis and induce spontaneous necrosis of the affected liver cells in man. Again, the utilization of glucose by Plasmodium and the resulting formation of lactic acid with the consequential release of free radicals into the extracellular compartment of the liver cause bleb of the hepatocyte plasma membrane. In the liver sections observed, the absence of Kupffer cell hyperplasia and haemosiderosis in the CYP/XA-treated groups other than the lowest combination dose could partly be due to the significant reduction of parasitaemia by the treatment regimen.

The spleen which is the site for the breakdown of worn-out red blood cells in *P. berghei* infection and subsequently storing the iron they contain apparently showed no loss of cellular architecture. Apart from the lowest dose, all the other treatment doses of the CYP + XA combination treatment showed mild reduction of these pathologies caused by the *P. berghei* parasite in the tissues of the spleen compared to the vehicle-treated mice. Histological analysis of the kidney showed that the cryptolepine–xylopic acid combination drug was unable to restore the damage caused by the infiltration of perivascular interstitial mononuclear cells which are located between tubules of the nephron.

Histopathological analysis of the testes of animals treated with the lowest dose of CYP + XA showed normal morphological appearance with seminiferous tubules with all the various stages of spermatogenesis. Anti-androgenic and spermatotoxicity of both cryptolepine and xylopic acid have been previously suggested at higher doses [27, 28] and it was very apparent in this study.

## Conclusion

Results from this study confirms the antiplasmodial effects of cryptolepine and xylopic acid and further demonstrate that co-administration of the two drug candidates exhibit a synergistic interaction with minimal toxicological effects in the testes during malaria infections in rats at relatively higher doses. Further detailed toxicity studies using animal models and human tissues to enable the possible use of these drugs in human populations is recommended.

## Abbreviations

CYP: cryptolepine
XA: xylopic acid
ED_50_: median effective dose
Z_add_: theoretical ED_50_
Z_exp_: experimental ED_50_
ART: artemether
LUM: lumefantrine
